# Targeting synaptic plasticity to bridge translational gaps in sepsis-associated encephalopathy

**DOI:** 10.3389/fnagi.2025.1616736

**Published:** 2025-06-10

**Authors:** Koji Hoshino

**Affiliations:** Department of Anesthesiology, Hokkaido University Hospital, Sapporo, Japan

**Keywords:** sepsis-associated encephalopathy, synaptic plasticity, long-term potentiation, hippocampus, neuroinflammation

## Abstract

Sepsis-associated encephalopathy (SAE) is a frequent yet underrecognized complication of sepsis that significantly contributes to long-term cognitive dysfunction in survivors. Despite advances in sepsis management, there is currently no established therapy targeting SAE, and translational gaps between basic and clinical research persist. Rodent models of sepsis suffer from variability in immune responses and poor translational fidelity. Moreover, behavioral tests commonly used to assess cognition in animal models are often confounded by sepsis-induced sickness behaviors and depression-like phenotypes, especially during the acute phase. Given these limitations, targeting synaptic plasticity—both mechanistically and therapeutically—has emerged as a promising approach. Accumulating evidence indicates that SAE arises from neuroinflammation triggered by systemic inflammation, in which activated microglia and subsequent cytokine signaling contribute to neuronal dysfunction and lead to impaired hippocampal long-term potentiation (LTP), a fundamental mechanism of learning and memory. Importantly, electrophysiological studies have shown that LTP impairment occurs within hours to days after sepsis onset, highlighting its potential as an early and sensitive biomarker for SAE. Recent experimental interventions, including low-intensity exercise, environmental enrichment, and modulation of gut microbiota, have shown beneficial effects on SAE. These findings underscore the need for integrative, multimodal strategies that address the complex pathophysiology of SAE. Synaptic plasticity, particularly LTP, may serve not only as a functional readout of neuroinflammatory damage but also as a modifiable target for early intervention. This review highlights the translational challenges in current SAE research and advocates for a paradigm shift toward mechanism-driven and plasticity-focused therapeutic development.

## 1 Introduction

Sepsis remains a global health burden, accounting for nearly 20% of all deaths worldwide, although its incidence and mortality have declined substantially over the past 25 years (Rudd et al., 2020). The link between sepsis and cognitive dysfunction has long been recognized. A historical cohort study involving approximately one million individuals aged 65 years or older in the United Kingdom reported that sepsis nearly doubles the risk of subsequent dementia (Muzambi et al., 2021). Sepsis-associated encephalopathy (SAE) refers to brain dysfunction occurring in the context of sepsis, without direct central nervous system infection. There is currently no standardized diagnostic criterion for SAE. Eidelman et al. (1996) first proposed the utility of the Glasgow Coma Scale (GCS) in defining SAE. Since then, numerous studies have defined SAE using criteria such as impaired consciousness with a GCS score <15 (typically <13–14) or the presence of delirium as assessed by tools like the Confusion Assessment Method for the Intensive Care Unit (CAM-ICU; Thy et al., 2025).

SAE presents with a broad clinical spectrum, ranging from mild disorientation to coma, and affects nearly half of all septic patients. Even mild consciousness disturbances (e.g., GCS 13–14) are associated with increased mortality (Sonneville et al., 2017). Beyond its acute impact, SAE can lead to persistent cognitive impairments. Although more than 30% of patients experience resolution of cognitive symptoms within 1 month of sepsis onset, some individuals exhibit long-term deficits (Manabe and Heneka, 2021). As a result, sepsis management is no longer focused solely on survival alone, and SAE must be recognized as a worthy therapeutic target. Despite numerous clinical trials, including those with vitamin C and thiamine (Park et al., 2020), no effective treatments have been established, and basic research on SAE has not yet been translated into clinical practice. This review aims to delineate the challenges faced in basic SAE research and explore future directions that may facilitate therapeutic development.

## 2 Pathophysiology of SAE

In the past 10–15 years, major progress has been made in elucidating the pathophysiological mechanisms of SAE. It is now widely accepted that systemic inflammation initiates neuroinflammation, which subsequently leads to cognitive impairment (Denver and Cunningham, 2025). There are four principal pathways through which peripheral inflammation induces neuroinflammation (Dantzer et al., 2008; Widmann and Heneka, 2014; Barbosa-Silva et al., 2021): (1) Increased blood-brain barrier (BBB) permeability, allowing cytokines and immune cells to infiltrate the brain parenchyma. BBB disruption is associated with dysfunction of the glial vascular unit, involving astrocytes, microglia, endothelial cells, and pericytes (Hu et al., 2025). (2) Interleukin (IL)-1 receptor-mediated signaling from perivascular macrophages and endothelial cells initiates localized inflammatory responses. (3) Humoral signaling through circumventricular organs, which lack a complete BBB. Macrophage-like cells expressing toll-like receptors release cytokines that diffuse into the brain via volume diffusion or active transport. (4) Neural signaling via the vagus and trigeminal nerves, which detect peripheral IL-1β and transmit signals to the brain.

All these pathways converge on microglial activation, which sustains the neuroinflammatory state. Downstream consequences of activated microglia include enhanced expression of pro-inflammatory cytokines, mitochondrial dysfunction, and oxidative stress. These factors contribute to the formation of the nucleotide-binding oligomerization domain, leucine-rich repeat and pyrin domain-containing protein 3 (NLRP3) inflammasome, leading to the production of mature IL-1β (Manabe and Heneka, 2021; Moraes et al., 2021). Recent studies suggest that persistent activation of the NLRP3-IL-1β pathway may underlie chronic SAE (Beyer et al., 2020; Zhao et al., 2020). Clinically, advanced age and pre-existing cognitive decline have been associated with prolonged SAE (Sonneville et al., 2017). Our group has demonstrated elevated hippocampal IL-1β levels during the acute phase of sepsis in senescence-accelerated mice, suggesting a role for the NLRP3-IL-1β pathway in the chronicity of SAE (Hoshino et al., 2021).

Recently, Gao et al. (2024) reported that N-acetyltransferase 10 (NAT10) is upregulated in hippocampal neurons of the dentate gyrus as a downstream mechanism following microglial activation. NAT10 promotes the acetylation of gamma-aminobutyric acid B receptor 1 (GABA_B_R1) mRNA, enhancing its expression and increasing inhibitory synaptic currents, thereby contributing to impaired neurotransmission and learning deficits. Additionally, excessive microglia-mediated synaptic pruning has been reported. For instance, decreased hippocampal PSD-95 expression at 24 h post-lipopolysaccharide (LPS) injection (Song et al., 2020) and reduced neocortical spine density at 8 weeks (Kondo et al., 2011) have been documented. These findings underscore the complex, multifactorial nature of SAE and support the need for multipronged therapeutic strategies.

## 3 Challenges in basic research on SAE

### 3.1 Limitations of rodent models in sepsis research

One persistent criticism is that basic research on sepsis has failed to yield translational benefits (Cavaillon et al., 2020). Rodent models are limited by their intrinsic resistance to endotoxins and robust recovery from infections (Warren et al., 2010). Moreover, genetic differences among inbred strains lead to variable immune responses (Sellers et al., 2012). While humanized mice have been proposed as a potential solution (Laudanski, 2021), their complexity and cost hinder widespread adoption.

Common sepsis models include bacterial infection, intraperitoneal injection of LPS (endotoxemia model), cecal ligation and puncture (CLP), and cecal slurry. A recently proposed alternative is the fecal suspension and intraperitoneal injection model (Tsuchida et al., 2022). Each has strengths and limitations (Cai et al., 2023). Endotoxemia models poorly replicate clinical sepsis, and LPS results vary depending on type and lot (Beyer et al., 2020). Even CLP, the so-called “gold standard,” is subject to variability based on cecum size, needle gauge, extruded fecal volume, and fat handling (Buras et al., 2005; Niiyama et al., 2016). As Gonnert et al. (2011) emphasized, standardized models using clinical severity scores are essential for reproducible original laboratory research.

### 3.2 Limitations of behavioral testing in sepsis animal model

In contrast to humans, encephalopathy in animals cannot be assessed using GCS or CAM-ICU. As a result, behavioral tests evaluating cognition—such as learning and memory—are employed. The hippocampus is considered the most vulnerable brain region in sepsis (Semmler et al., 2005), and hippocampus-dependent tasks are preferred. However, contextual fear conditioning and the Morris water maze require equivalent baseline locomotor activity between groups—a condition not met in sepsis models. Sepsis induces profound behavioral alterations, including sickness behavior during the acute phase, marked by reduced activity, food intake, weight loss, and sleep disturbance. This is followed by depression-like behavior, attributed to cytokine-induced activation of indoleamine 2,3-dioxygenase (IDO), leading to anhedonia and helplessness (Dantzer et al., 2008; Pereira De Souza Goldim et al., 2020). These overlapping behavioral states complicate data interpretation (Dantzer et al., 2008). Indeed, even endotoxemia models show that such behaviors persist for up to 14 days after intraperitoneal LPS injections (Sohroforouzani et al., 2022), corresponding to the clinical ICU phase, during which behavioral testing may not reliably reflect cognitive function. Furthermore, general limitations of behavioral tests include experimenter bias, stress induction, low ecological-validity, and circadian mismatches (Lang et al., 2023). When coupled with sepsis-specific behavioral abnormalities, these factors introduce substantial confounds.

## 4 SAE and the pathophysiological role of synaptic plasticity

### 4.1 The impact of SAE on long-term potentiation

Neuroinflammation resulting from sepsis leads to various forms of synaptic dysfunction, including neurotransmitter imbalance, synaptic deficiency, myelin damage, and inhibition of synaptic plasticity (Tang et al., 2022; Yang et al., 2025). Synaptic plasticity is broadly categorized into structural and functional types. Among the latter, long-term potentiation (LTP)—first described by Bliss and Collingridge (1993)—is regarded as a cellular substrate for learning and memory (Takeuchi et al., 2014).

Historically, SAE research relied heavily on behavioral assessments. However, due to limitations in such tests during the acute phase of sepsis, electrophysiological approaches have gained traction. In 2000, Vereker et al. (2000) demonstrated impaired LTP in the hippocampal Schaffer collateral (SC)–CA1 pathway 3 h after intraperitoneal LPS injection in rats. Imamura et al. (2011) later reported reduced LTP in the CA1 region 24 h post-CLP.

Table 1 summarizes key studies evaluating hippocampal LTP in rodent SAE models. Many studies report reduced LTP during the acute phase (3 h−14 days post-insult), highlighting its utility as a surrogate marker for SAE. However, outcomes vary depending on stimulation protocols and animal age. Chapman et al. (2010) found no reduction in early-phase LTP (E-LTP) following 100 Hz stimulation in either young (3 months) or aged (24 months) rats 4–5 days after intraperitoneal *E. coli* infection. Interestingly, only aged rats exhibited reduced late-phase LTP (L-LTP) in response to theta-burst stimulation (TBS) 8 days after *E. coli* infection (Tanaka et al., 2018), suggesting that the impairment is both age-specific and dependent on the stimulation protocol. Kakizaki et al. (2023) showed that immature rats (4–5 weeks old) subjected to CLP displayed no significant reduction in E-LTP at 8 or 16 h post-insult. These findings emphasize the need to account for stimulation protocol and animal age when interpreting LTP outcomes in SAE models. Although most studies summarized in Table 1 have been conducted exclusively in male rodents, recent evidence indicates that hippocampal synaptic plasticity exhibits sex-dependent mechanisms. For instance, N-methyl-D-aspartate (NMDA) receptor contributions to LTP and spatial memory differ between sexes even in juvenile animals (Narattil and Maroun, 2024), and estrogen receptor signaling critically regulates LTP in females (Wang et al., 2018). Future studies should address sex as a biological variable to enhance translational relevance.

**Table 1:** Overview of experimental studies on sepsis models and hippocampal LTP (chronological order).

**Animal**	**Model**	**Timing of LTP assessment after sepsis induction**	**Stimulation protocol and recording site**	**LTP changes**	**References**
Wistar rat (250–300 g)	LPS i.p.	3 h	HFS/PP–DG	E-LTP impaired	Vereker et al., 2000
Fischer/Brown Norway rat (3 and 24 months)	*E. coli* i.p.	4–5 days	TBS or HFS/SC–CA1	L-LTP impaired (TBS only)	Chapman et al., 2010
C57BL/6J (8 weeks)	CLP	24 h	HFS/PP–DG	E-LTP impaired	Imamura et al., 2011
C57BL/6/SvEv129 (3–4 months)	LPS i.p.	3 h	TBS/SC–CA1	E-LTP impaired	Wang et al., 2012
Wistar rat (250–300 g)	LPS i.p.	6 h	HFS/SC–CA1	E-LTP impaired	Zivkovic et al., 2015
C57BL/6J (6 weeks)	CLP	24 h	TBS/SC–CA1	E-LTP impaired	Hoshino et al., 2017b
Fischer/Brown Norway rat (3 and 24 months)	*E. coli* i.p.	8 days	TBS/SC–CA1	L-LTP impaired (aged rats only)	Tanaka et al., 2018
C57BL/6 (8–12 weeks)	LPS i.p. and CLP	7 days	TBS/SC–CA1	E-LTP impaired (both LPS and CLP)	Hippensteel et al., 2019
BALB/c (8–12 weeks)	CLP	24 h	TBS/PP–DG	E-LTP impaired	Xu et al., 2019
C57BL/6J (19 months)	LPS i.p. (*E. coli* or *S. typhimurium*)	3 months	TBS/SC–CA1	E-LTP impaired (*S. typhimurium* only)	Beyer et al., 2020
Senescence-Accelerated Mouse (SAM) R1 and P8 (6 months)	CLP	24 h	HFS/SC–CA1	E-LTP impaired (SAMR1 only)	Hoshino et al., 2021
SD rat (8 weeks)	CLP	6 days	HFS/SC–CA1	E-LTP impaired	Qin et al., 2021
SD rat (4–5 weeks)	CLP	8 and 16 h	TBS/SC–CA1	No LTP impairment	Kakizaki et al., 2023
C57BL/6J (6–8 weeks)	LPS i.p.	3 days	HFS/SC–CA1	E-LTP impaired	Yin et al., 2023a
C57BL/6J (10–12 weeks)	CLP	14–16 days	HFS/SC–CA1	E-LTP impaired	Yin et al., 2023b
C57BL/6J (unknown age)	Peritoneal contamination and infection	8 weeks	TBS/SC–CA1	E-LTP impaired	Grünewald et al., 2024
C57BL/6J (10–12 weeks)	LPS i.p.	6 days	HFS/SC–CA1	E-LTP impaired	Lv et al., 2024
C57BL/6J (8–12 weeks)	CLP	7 days	TBS/SC–CA1	L-LTP impaired	Soejima et al., 2025

LTP, long-term potentiation; E-LTP, early-phase LTP; L-LTP, late-phase LTP; LPS, lipopolysaccharide; CLP, cecal ligation and puncture; PP, perforant path; DG, dentate gyrus; SC, Schaffer collateral; HFS, high-frequency stimulation; TBS, theta-burst stimulation.

### 4.2 Mechanisms of LTP impairment in SAE

In NMDA receptor-dependent LTP, as seen in hippocampal SC–CA1 and perforant path (PP)–dentate gyrus (DG) pathways, calcium influx via NMDA receptors—elicited by TBS or high-frequency stimulation (HFS)—activates calcium–calmodulin-dependent protein kinase II (CaMKII) and enhances alpha-amino-3-hydroxy-5-methyl-4-isoxazolepropionic acid receptor (AMPAR) trafficking, thereby potentiating synaptic transmission (Nicoll and Roche, 2013).

A key mechanism by which SAE impairs LTP involves the activation of the NLRP3–IL-1β pathway in microglia. Although physiological levels of IL-1β are thought to be important for the maintenance of hippocampal LTP (Ross et al., 2003), its overexpression has long been known to disrupt hippocampal LTP (Bellinger et al., 1993), particularly the NMDA receptor-dependent form (Hoshino et al., 2017a). This impairment is mediated via activation of p38 mitogen-activated protein kinase (MAPK; Coogan et al., 1999). IL-1β also promotes the neuronal surface expression of GABA_A_ receptors—especially those containing the α5 subunit—leading to increased tonic inhibitory currents and further LTP impairment through p38 MAPK-dependent mechanisms (Serantes et al., 2006; Wang et al., 2012). Additionally, IL-1β suppresses NMDA-induced outward currents via p38 MAPK (Zhang et al., 2010), offering another potential mechanism of LTP disruption.

Furthermore, microglia-driven synaptic pruning, discussed earlier in the context of SAE pathophysiology, may also impair hippocampal plasticity, warranting further investigation (Yang et al., 2025).

### 4.3 LTP as a therapeutic target in SAE

Grünewald et al. (2024) reported significant downregulation of *Arc/Arg3.1*—a master regulator of synaptic plasticity (Shepherd and Bear, 2011)—in the hippocampus of mice subjected to peritoneal contamination and infection. Hippocampal overexpression of *Arc* with adeno-associated virus vectors restored impaired E-LTP. Similarly, environmental enrichment during the post-septic period enhanced hippocampal *Arc* expression via the brain-derived neurotrophic factor (BDNF)/TrkB pathway, rescuing LTP and spatial memory. These findings suggest that directly targeting impaired LTP could offer therapeutic benefits.

In clinical studies, cerebrospinal fluid (CSF) from infected patients with delirium showed reduced levels of proteins involved in synapse formation and function, compared to non-delirious patients (Peters Van Ton et al., 2020), lending further support to this hypothesis. Moreover, postoperative cognitive dysfunction—believed to share pathophysiological features with SAE—has been linked to impaired E-LTP to L-LTP transformation mediated by hippocampal BDNF downregulation (Liu et al., 2025), underscoring the growing relevance of LTP-focused interventions. To date, no clinical studies have directly examined the relationship between hippocampal LTP and standard cognitive assessments such as the Mini-Mental State Examination (MMSE) or Montreal Cognitive Assessment (MoCA) in patients with sepsis or SAE. However, indirect evidence from other neurological conditions supports a potential link between LTP-related plasticity and cognitive performance. For instance, in patients with multiple sclerosis, reduced transcranial magnetic stimulation (TMS)-induced cortical plasticity—considered a human proxy for LTP—was associated with both lower CSF amyloid-β_1 − 42_ levels and poorer attention and concentration scores on cognitive tests (Mori et al., 2011). Although these data reflect neocortical rather than hippocampal plasticity, they suggest that LTP-like measures may serve as functional indicators of cognitive integrity. Given the role of hippocampal LTP in memory formation, future studies are warranted to determine whether electrophysiological markers of hippocampal plasticity in animal models of SAE correspond to clinical memory assessments such as MMSE or MoCA in human patients. Establishing such links would enhance the translational value of LTP as a surrogate marker and may provide a measurable endpoint for therapeutic trials targeting cognitive dysfunction in SAE.

## 5 Discussion

Numerous compounds have demonstrated therapeutic potential in preclinical models of SAE (Barichello et al., 2019; Catarina et al., 2021). Nevertheless, no pharmacologic agent has yet shown clinical efficacy. The use of standardized, reproducible animal models with clinical severity scoring systems is essential. Given the behavioral confounds during the acute-to-subacute phase of sepsis, LTP offers a reliable and interpretable surrogate marker. As SAE pathophysiology is inherently multifaceted, monotherapies are unlikely to effectively address its complexity. Multimodal interventions—including pharmacologic, nutritional, and physical therapies typical of intensive care—should be integrated into future preclinical research to foster successful translation to clinical care. Indeed, previous studies have shown that environmental enrichment following endotoxemia improved spatial learning in mice (Keymoradzadeh et al., 2020), and that low-intensity exercise during the acute phase of sepsis restored hippocampal synaptic plasticity (Soejima et al., 2025). Moreover, recent research has also highlighted a potential link between sepsis-induced gut dysbiosis and the development of SAE (Fang et al., 2022). In translating therapeutic interventions such as environmental enrichment and low-intensity exercise from rodent models to clinical practice, practical barriers remain. Environmental enrichment paradigms in rodents typically involve access to toys, running wheels, and group housing, which do not directly map onto human hospital settings. For example, in stroke care, enriched environments have included cognitive stimulation through music, games, and communal spaces, yet their definitions and applications remain inconsistent and context-dependent (Anåker et al., 2024). To date, there have been no clinical trials assessing environmental enrichment in patients with sepsis or SAE. This highlights the need for research to define and translate rodent-based enrichment paradigms into feasible and effective interventions for human patients. Similarly, early rehabilitation has shown promise in improving physical function and inducing systemic anti-inflammatory effects in sepsis patients (Kayambu et al., 2015). Rodent studies often employ short-term, low-intensity treadmill protocols, but their clinical counterparts must account for patient variability, resource limitations, and safety considerations. Such translational gaps call for research frameworks that integrate both experimental rigor and clinical practicality. Future efforts should focus on developing mechanistically informed, multimodal strategies—guided by insights into synaptic plasticity—that can serve both as measurable biomarkers and modifiable targets for restoring cognitive function.
